# Feasibility of 4 patient-reported outcome measures in a registry setting: A cross-sectional study of 6,000 patients from the Danish Hip Arthroplasty Registry

**DOI:** 10.3109/17453674.2012.702390

**Published:** 2012-08-25

**Authors:** Aksel Paulsen, Alma B Pedersen, Søren Overgaard, Ewa M Roos

**Affiliations:** ^1^Department of Orthopaedic Surgery and Traumatology, Odense University Hospital, Institute of Clinical Research, University of Southern Denmark, Odense; ^2^Department of Clinical Epidemiology, Aarhus University Hospital, Aarhus; ^3^Research Unit for Musculoskeletal Function and Physiotherapy, Institute of Sports Science and Clinical Biomechanics, University of Southern Denmark, Odense, Denmark

## Abstract

**Background and purpose:**

Feasibility is an important parameter when choosing which patient-reported outcomes (PRO) to use in a study. We assessed the feasibility of PROs in a hip registry setting.

**Methods:**

Primary total hip arthroplasty (THA) patients (n = 5,747) who had been operated on 1–2, 5–6, or 10–11 years previously were randomly selected from the Danish Hip Arthroplasty Register and sent 2 PRO questionnaires: 1 generic (EuroQoL-5D or SF-12 health survey) and 1 disease-specific (hip dysfunction and osteoarthritis outcome score (HOOS) or Oxford 12-item hip score). We compared response rates, floor and ceiling effects, missing items, and the need for manual validation of forms.

**Results:**

4,784 patients (mean age 71 years, 57% females) were included (83%). The response rates ranged from 82–84%. Statistically significantly different floor and ceiling effects ranged from 0% to 0.5% and from 6.1% to 46%, respectively. Missing items ranged from 1.2% to 3.4%, and 0.8–4.3% required manual validation (p < 0.009). A hypothetical repeat study found that group sizes from 51 to 1,566 are needed for subgroup analysis, depending on descriptive factor and choice of PRO.

**Interpretation:**

All 4 PROs fulfilled a priori set criteria, with the exception of ceiling effects. The high ceiling effects were attributed to postoperative administration and good outcome for THA. We conclude that all 4 PROs are appropriate for administration in a hip registry.

In the past few decades, several new patient-reported outcomes (PROs) on hip function have been introduced for use in research and clinical practice. The Department of Health in the UK now requires PRO data for all National Health Service patients in England and Wales before and after total joint arthroplasty (Devlin et al. 2010), and PROs have also been introduced in other national hip arthroplasty registries (Rolfson 2010, Rothwell et al. 2010, Rolfson et al. 2011). A PRO is not valid per se, but has to be validated in the context of interest. In earlier reports, the feasibility of PROs in a joint registry setting was defined as “the average usable response rate for a questionnaire in a postal survey” (Dunbar 2001). Since then, it has been clear that many other factors are important and should be considered when introducing a PRO into a registry setting. There has been a limited amount of research on this broader definition of feasibility, and there has been little research in which specific PROs in registry settings have been compared.

We compared the feasibility of 4 PROs: 2 generic (EuroQoL-5D (EQ-5D) and the SF-12 health survey) and 2 disease-specific (the hip dysfunction and osteoarthritis outcome score (HOOS) and the Oxford 12-item hip score (OHS) by testing response rates, floor and ceiling effects, missing items, and need for manual validation of forms in patients registered in the Danish Hip Arthroplasty Registry (DHR). We also calculated the number of patients needed for each PRO to discriminate between subgroups of age, sex, diagnosis, and prosthesis type in a hypothetical repeat study.

## Patients and methods

### Generic outcome measures

EQ-5D (The EuroQol Group 1990) is a generic measure of health-related quality of life (HRQoL), which has been validated in total hip arthroplasty (THA) patients (Dawson et al. 2001) and rheumatoid arthritis patients (Linde 2009). We used a Danish value set (Wittrup-Jensen et al. 2009) when computing the index.

SF-12 is a generic measure of health status (Ware et al. 1996) that has been validated in OA patients (Gandhi et al. 2001). The SF-12 gives 2 summary scores: a physical component summary (PCS) and a mental component summary (MCS), by computation with a standardized scoring algorithm. PCS and MCS were treated as one variable in the analyses since they are derived from the same items but with different weightings, due to dependence.

### Disease-specific outcome measures

The HOOS includes 5 subscales: Pain, Other Symptoms, Function in Daily Living, Function in Sport and Recreation, and Hip-related Quality of Life. The HOOS Physical Function short form (HOOS-PS) is a 5-item short version derived from the 2 HOOS subscales: Function in Daily Living and Function in Sport and Recreation. The HOOS-PS has recently been validated for THA (Davis et al. 2009). For the purpose of our study, we used 3 different HOOS subscales—HOOS Pain, HOOS Physical Function short form (HOOS-PS), and HOOS Hip-related Quality of Life (QoL)—to measure pain, physical function including daily activities and more strenuous physical activities, and hip-related quality of life. To keep a low number of items, we included only these 3 subscales. A score of 100 indicates no problems and 0 indicates severe problems.

The OHS (Dawson et al. 1996) is a 12-item PRO developed for patients undergoing THA, and focuses on activities of daily living. A summed score of between 0 and 48 is calculated, with 48 indicating the best possible result. The OHS has been shown to be consistent, reliable, valid, and sensitive to clinical change following THA (Murray et al. 2007). As part of this project, the OHS has been translated from the English-language version into Danish and validated in accordance with the protocol for cross-cultural linguistic validation of PROs (Wild et al. 2005) and the user manual (Dawson et al. 2010).

### Data collection

We used a cross-sectional design, based on a cohort of patients registered in the DHR with primary THA as index operation. The DHR is a nationwide, population-based, clinical database of all primary THAs and revisions performed in Denmark since January 1995. From 1995 until 2010, 103,424 primary THAs and 16,524 revisions were recorded. The completeness of the DHR regarding primary THA is 96%, whereas the coverage (proportion of clinics reporting to the DHR) is 100% (Overgaard 2012).

A sample of 5,777 patients with primary THA who underwent surgery 1–2, 5–6, and 10–11 years previously were randomly selected, to obtain samples of short-, middle-, and long-term follow-up. We sampled from all patients over 18 years of age (approximately 1,900 patients for each year). We made sure that there was equal composition regarding age in the 3 groups. Patients who later had revision surgery, or contralateral THA following the index operation, were not excluded from the study.

Every patient received 2 different PROs, 1 generic and 1 disease-specific, in 4 groups of approximately 500 patients from each follow-up group (Figure). None of the groups received the same pair of PROs. Sample-size calculation showed that, assuming a risk of type I error of 0.05 (2-sided test) and a power of 80% to detect a relative risk of 2.0 for difference between the groups (i.e. response rate etc.), approximately 500 patients in each group would be needed.

**Figure:**
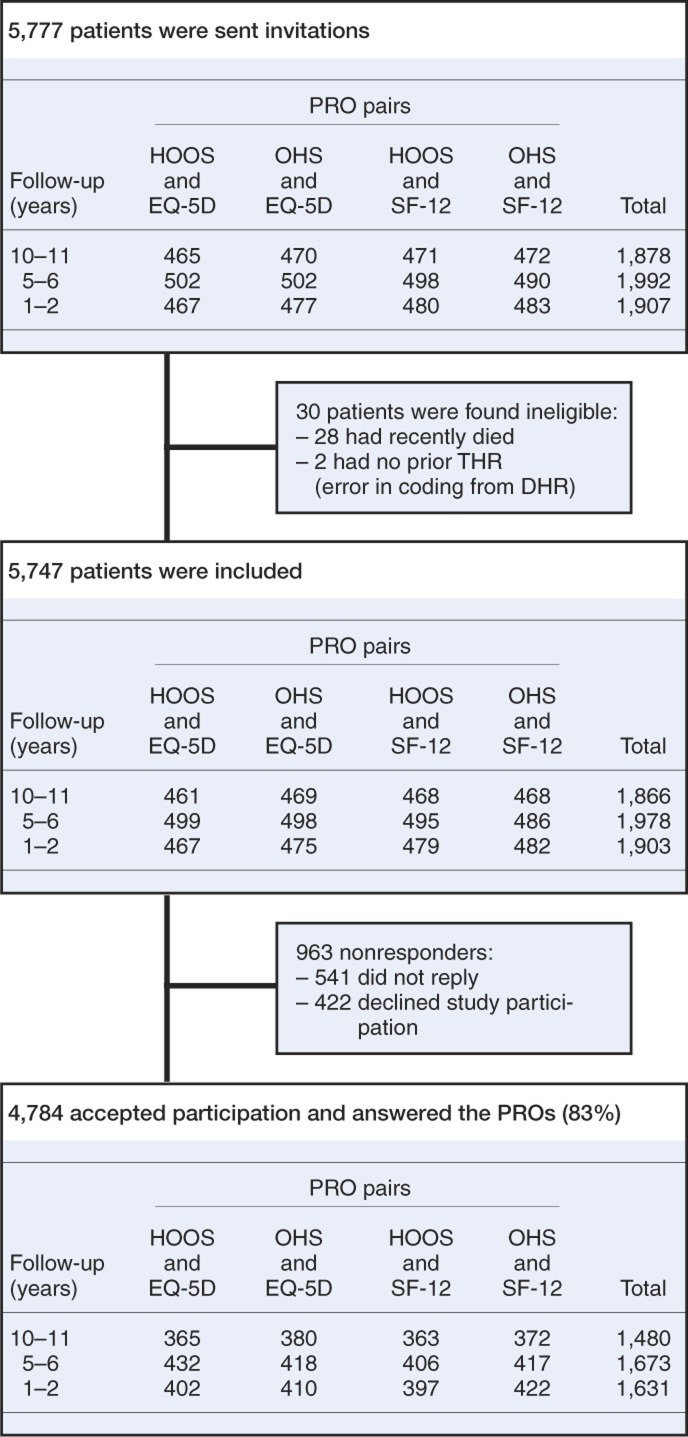
Patient flow chart. Each patient had a generic PRO (EQ-5D or SF-12) and a disease-specific PRO (HOOS or OHS) 1–2 years, 5–6 years, or 10–11 years after primary surgery.

The PROs were mailed in paper form to the patients by regular post including a stamped, addressed envelope for return. Up to 2 reminder letters were sent. All returned PRO forms were scanned electronically using a validated automated forms-processing technique (Paulsen et al. 2012). Manual validation was conducted when our automated forms-processing system could not interpret a PRO answer. Patients were classified as responders (those who accepted participation and answered the PROs) and non-responders (those who declined to participate or simply did not reply to the invitation letter) (Figure 1).

### Feasibility criteria

The PROs were assessed in relation to the following for feasibility: response rate, floor and ceiling effects, missing items, and the need for manual validation of the scanned PROs. Response rate was determined as the percentage of patients who accepted participation and answered the PROs out of the total number of patients who were sent the PRO. Floor and ceiling effects indicate the percentage of patients for whom it would not be possible to measure a meaningful deterioration or improvement of their condition because they are already at the extreme end of the PRO. Floor and ceiling effects were calculated as the percentage of patients with the lowest or highest possible sum score (for example, a total score of 0 or 48 for the OHS) out of the total number of patients who answered each PRO.

Concerning missing items, we examined both missing items and discarded PRO subscales. The proportion of items missing was defined as the percentage of items that were missing out of the total number of items received for each PRO. The missing items were treated in accordance with the manual of the PRO in question in order to calculate the total score for the different PROs (Appendix Table 1, see Supplementary data). Discarded PRO subscales were defined as the percentage of PRO subscales with too many items missing to give valid information (as defined by the manual or guide for each PRO) out of the total number of subscales received for each PRO.

**Table 1.** Patient characteristics of responders and non-responders

Descriptive statistics	Responder	Non-responders	p-value
Population (n)	4,784	963	
Percent of total	83	17	
Age **^a^** (median)	73	78	< 0.001
Range (years)	19–102	18–101	
18–50 years (%)	5.4	6.0	0.5
50–70 years (%)	36	26	< 0.001
> 70 years (%)	59	68	< 0.001
Female sex (n)	2,750	635	
Percent	58	66	< 0.001
Diagnosis (%) **^b^**			
Idiopathic osteoarthritis	84	72	< 0.001
Low-impact fractures	7.9	19	0.01
Childhood diseases	4.3	3.4	0.2
Other arthritis	2.5	4.0	0.8
High-impact injuries	1.0	0.9	< 0.001
Prosthesis design (%)			
Uncemented	44	33	< 0.001
Cemented	31	38	< 0.001
Hybrid	25	29	0.03

**^a^** Age of patients on date of sending PRO.

**^b^** Idiopathic OA, other arthritis (rheumatoid arthritis, morbis Bechterew, other arthritis), childhood diseases (congenital hip dislocation, morbis Calvé-Legg-Perthes, epiphysiolysis, acetabular dysplasia), high-impact injuries (fracture of acetabulum, traumatic hip dislocation) and low-impact fractures (fresh fracture of proximal femur, late sequelae from fracture of proximal femur). The diagnosis atraumatic necrosis of the femoral head (2.5%) and other diagnoses (0.6%) are not shown in this table.

The need for manual validation was assessed as both the proportion of questionnaires requiring manual validation and the proportion of items validated, to take into consideration the different number of items in the PROs. The proportion of questionnaires requiring manual validation was defined as the percentage of questionnaires in which manual validation was required out of the total number of questionnaires of a particular kind received. The proportion of items requiring manual validation was defined as the percentage of items in each questionnaire that were manually validated out of the total number of items in a questionnaire.

### Statistics

Response rate, floor and ceiling effects, missing items, and the need for manual validation were calculated as proportions with 95% confidence intervals (CIs). We used a chi-squared test to compare the proportions. Any p-value of less than 0.05 was considered significant. A priori, we had defined cut-offs for all 5 criteria in order to identify PROs that were feasible for use in registry settings: overall response rate over 80%, floor and ceiling effects less than 15%, a proportion of items missing of less than 5%, and a proportion of items needing manual validation of less than 5%.

Logistic regression was used to compare overall feasibility criteria between different PROs, adjusting for age (< 50, 50–70, and > 70 years), sex, primary hip diagnosis (idiopathic OA, inflammatory arthritis, childhood diseases, high-impact injuries, and low-impact fractures) and prosthesis type (uncemented, cemented, or hybrid). Odds ratios with 95% CIs were calculated.

The abilities of different PRO subscales to discriminate between age and sex groups, diagnostic groups, and prosthesis types were studied using analysis of variance. The hypothetical number of subjects needed to find the significant difference in mean value of a PRO between groups (assuming a significance level of 5% and a power of 85% to detect differences between the actual groups) was estimated for each PRO subscale with sample-size calculations or with power calculations and simulated ANOVA F tests, depending on the number of groups. We used STATA software version 10.1 and 11.0 for all the statistical analyses.

### Ethics

The study was approved by the Danish Data Protection Agency (journal number 2008-41-2593), the Danish National Board of Health, and the DHR. The study was carried out in accordance with the World Medical Association Declaration of Helsinki. All patients gave their informed written consent before participation in the study.

## Results

### Description of the study population

4,784 of 5,747 patients (83%) were included in the analysis (Figure 1). Non-responders were significantly older than responders (median age 78 years vs. 73 years (p < 0.001)) and were more often females (66% vs. 58% (p < 0.001)) (Table 1). There were no significant differences regarding number of patients in different age groups, sex, diagnosis group, or type of prosthesis (p = 0.4–1.0). The mean scores for the 4 different PROs (for the total population) are given in Table 2.

**Table 2.** PRO scores for the total population

PRO	Mean (95% CI)
HOOS (n = 2,365)	
HOOS Pain	88 (86–88.8)
HOOS PS	83 (81–84.0)
HOOS QoL	77 (75–78.4)
OHS (n = 2,419)	39 (38–39.6)
SF-12 (n = 2,377)	
SF-12 PCS	35 (34–35)
SF-12 MCS	49 (48–50)
EQ-5D (n = 2,407)	
EQ-5D Index	0.84 (0.83–0.86)
EQ-VAS	80 (78–81)

### Response rate

All PROs fulfilled our criteria of an overall response rate of over 80% (Table 3). The response rates for the disease-specific PROs were 82.4% for HOOS and 84.1% for OHS (p = 0.1). Multiple regression analyses adjusted for age, sex, diagnosis, and type of prosthesis showed no overall difference in the response rate for HOOS and OHS (adjusted OR = 0.90, CI: 0.78–1.04). The response rates for the generic PROs were 82.6% for SF-12 and 83.9% for EQ-5D (p = 0.2). The overall adjusted OR for response rate was 1.12 (CI: 0.97–1.30). Separate multivariate analyses of differences in response rate for disease-specific PROs and generic PROs showed similar results for females and for different age groups. However, males who had received the EQ-5D responded more often than males who had received the SF-12 (adjusted OR = 1.4, CI: 1.1–1.8).

**Table 3.** Overall results

	Specific PROs					Generic PROs				
	HOOS n = 2,365			OHS n = 2,419		SF-12 n = 2,377		EQ-5D n = 2,407		
	Pain	PS	QoL		P-value	PCS	MCS	EQ-5D Index	EQ-VAS	P-value
Response rate ^a^		82		84		83		84		
		(81–84)		(83–85)	0.100	(81–84)		(83–85)		0.2
Floor effect ^b^	0.1	0.1	0.5	0.0	< 0.001	0.1	0.1	0.0	0.3	0.03
		(0–0.2)	(0–0.3)	(0.3–0.8)		(0–0.3)	(0–0.3)		(0.1–0.5)	0.3
Ceiling effect ^b^	37	31	31	20	< 0.001	6.1	6.1	46	12	< 0.001
	(35–39)	(29–33)	(29–32)	(19–22)		(5.1–7.0)	(5.1–7.0)	(44–48)	(11–14)	
Proportion of items missing ^c^		3.4		1.2	< 0.001	2.3		1.9		0.009
		(3.2–3.5)		(1.0–1.3)		(2.1–2.5)		(1.7–2.2)		
Discarded PRO subscales ^b^	3.0	2.7	1.9	1.2	< 0.001	2.3	2.3	3.2	5.5	< 0.001
	(2.4–3.7)	(2.1–3.4)	(1.3–2.5)	(0.8–1.7)		(1.7–2.9)	(1.7–2.9)	(2.5–3.9)	(4.6–6.4)	
Proportion of items validated ^c^		0.9		1.5	< 0.001	0.8		4.3		< 0.001
		(0.8–1.0)		(1.4–1.7)		(0.7–1.0)		(4.0–4.6)		
Proportion of PROs requiring manual validation ^a^	7.8		7.2	0.4	7.7		22		< 0.001
		(6.7–8.9)		(6.2–8.2)		(6.7–8.8)		(20–23)		

Response rate defined as percentage that accepted participation and answered the PROs, out of the total number. Floor effect defined as percentage with worst possible outcome, out of total number. Ceiling effect defined as percentage with best possible outcome, out of total number.

^**a**^ Percentage of total number of PROs.

^**b**^ Percentage of total number of PRO subscales.

^**c**^ Percentage of total number of items.

### Floor and ceiling effects

All PROs fulfilled our criteria of a floor effect of less than 15%; the floor effect was 0.5% or less for the disease-specific PROs (p < 0.001) and less than 0.3% for the generic PROs (p = 0.03). However, neither the HOOS nor the OHS fulfilled our criteria of a ceiling effect of less than 15% (Table 3). Overall, HOOS Pain (adjusted OR = 2.4, CI: 2.1–2.7), HOOS PS (adjusted OR = 1.8, CI: 1.6–2.1), and HOOS QoL (adjusted OR = 1.8, CI: 1.6–2.0) had a higher ceiling effect than OHS. SF-12 PCS and MCS and the EQ-VAS fulfilled our criteria of a ceiling effect of less than 15%, while the EQ-5D Index had a high ceiling effect of 45.8% (p < 0.001). After adjustment, both EQ-5D Index (OR = 14, CI: 12–17) and the EQ-VAS (OR = 2.1, CI: 1.7–2.6) had higher ceiling effects than the SF-12.

### Missing items and discarded subscales

All PROs fulfilled our criteria of a proportion of items missing of less than 5% (Table 3). Females had a higher proportion of missing items than males, which was statistically significant for all subscales (p ≤ 0.001–0.4), except for HOOS QoL, OHS, and EQ-VAS (data not shown). The percentage of discarded PRO subscales, where a score could not be calculated due to too many missing items, was between 1.2% and 3.0% for disease-specific PROs (p < 0.001) and between 2.3% and 5.5% for generic PROs (p < 0.001). With multivariate analysis, we found a significantly higher risk of discarded PROs for female patients with HOOS Pain, HOOS PS, and HOOS Qol compared to patients with OHS. For the generic PROs, the EQ-5D Index and EQ-5D VAS had a higher risk of discarded questionnaires than SF-12 PCS/ MCS; adjusted OR for EQ-5D Index was 1.4 (CI: 1.0–2.1) and for EQ-VAS it was 2.6 (IC: 1.9–3.6).

### Manual validation

All PROs fulfilled our criteria of a proportion of items requiring manual validation of less than 5%. However, the proportion of questionnaires requiring manual validation exceeded 7% for all PROs (Table 3). For the generic PROs, 7.7% of the items in the SF-12 questionnaires required manual validation as compared to 21.8% in the EQ-5D questionnaires (p < 0.001).

### Discriminative ability

Group sizes from 51 to 1,566, depending on descriptive factors and choice of PRO, were needed for subgroup analysis (Table 4). OHS had the best discriminative ability—described by the hypothetical number of subjects needed to discriminate between groups in relation to gender (298 patients per group were needed to find a statistically significant difference in mean sum score). SF-12 PCS had the best discriminative ability in relation to diagnosis (51 patients per group were needed). EQ-VAS had the best discriminative ability regarding both age (where 270 patients per group were needed) and prostheses type (where 207 patients per group were needed).

**Table 4.** Discriminative ability; number of subjects needed per group

	Specific PROs				Generic PROs			
	HOOS			OHS	SF-12		EQ-5D	
	Pain	PS	QoL		PCS	MCS	EQ-5D Index	EQ-VAS
Diagnoses ^a^	116 ^e^	57 ^e^	115 ^e^	80 ^e^	51 ^e^	75 ^d^	107 ^e^	56 ^e^
Gender	502 ^e^	456 ^e^	760 ^e^	298 ^e^	1,886,596	2,736	414 ^e^	521 ^e^
Prosthesis groups ^b^	2,295	645 ^e^	10,308	795 ^e^	6,471	1,137	1,124 ^d^	207 ^e^
Age ^c^	15,461	814	685 ^d^	1,566 ^d^	384	361	3,360	270 ^e^

^**a**^ Idiopathic OA, other arthritis, childhood diseases, high-impact injuries, and low-impact fractures.

^**b**^ Hybrid prostheses, cemented and uncemented prostheses.

^**c**^ Less than 50 years old, 50–70 years old, and more than 70 years old.

^**d**^ p < 0.05.

^**e**^ p < 0.001.

## Discussion

The feasibility of a PRO is not absolute, but depends on the context in which it is being used. To our knowledge, this is the first feasibility study to compare commonly used disease-specific and generic PROs head-to-head in a hip registry setting. We found that all 4 PROs are feasible for use in a hip registry setting. Our feasibility criteria were response rate, floor and ceiling effects, missing items, and need for manual validation of the scanned PROs. A high response rate is important to ensure generalizability and to minimize selection bias. A response rate of 80% is usually considered to be sufficiently representative of the sample studied. We thus chose, a priori, this cut-off for the mailed patient-reported data used in the study. Much higher response rates are, however, achieved with regard to hard data entered into joint registries. For example, the DHR has a coverage of 96% (Overgaard 2012). These types of data collection differ with regard to the person providing the data (patient vs. health professional), ethics (patients are not legislated to provide data), and setting (in-hospital vs. home) and thus different response rates can be achieved.

Low floor and ceiling effects enable measurement of deterioration and improvement. The cut-offs were chosen based on previous findings (Terwee et al. 2007). A high percentage of missing items will make the PROs and sum scores less valid. The need for manual validation of the scanned PROs is an important indirect indication of the patient’s general ability to correctly fill in the PRO, and also provides information about the workload of the manual validation required. The complexity of a PRO or the lack of comprehensiveness can have an influence on response rate, the proportion of items missing, and the proportion of items requiring manual validation. Finally, the discriminative ability of each PRO gives a hypothetical number of subjects needed to discriminate between subgroups, and may contribute to the decision as to which PRO to use in further registry studies when subgroup analyses are of interest.

It is unclear whether follow-up time affects the response rate (Baker et al. 2007, Rothwell et al. 2010). We saw no difference in response rate with follow-up times ranging from 1 to 11 years, which supports the view that follow-up time is unrelated to response rate. To achieve our response rate, we used several strategies including using short questionnaires and sending out up to 2 reminders, as it is known that these strategies contribute to a higher response rate (Edwards et al. 2009). Due to the age of our patient population and their varying familiarity with computers and the internet, we used paper-based questionnaires sent by regular mail (Rolfson 2010).

The presence of floor and ceiling effects may influence the reliability, validity, and responsiveness of outcome measures. A worst or best score reported by 15% of the group studied is considered the maximum acceptable (Terwee et al. 2007). However, considering the good outcome of THA, low floor effects and high ceiling effects might be expected; therefore, the criterion of having the best possible score in less than 15% of patients following THA might be too restrictive. In support of this, others have reported a lower ceiling effect for the same PROs when administered preoperatively (Naal et al. 2009). A lower ceiling effect preoperatively than postoperatively is self-evident, and has been shown previously by others (Ostendorf et al. 2004). The lower ceiling effect in SF-12 PCS and SF-12 MCS may be due to computation of these subscales with a norm-based value set, which has also been shown by Linde (2009). Missing data reduce the quality of data. In a study of 3,156 RA patients, about 7% of patients were missing more than 20% of the items for SF-12 PCS, SF-12 MCS, and EQ-5D (Linde 2009). This high amount of missing items could in part be explained by a higher percentage of females included in that study (75–80%) than in the present study (58% females), as we found that females leave more unanswered items than males. We handled missing data in accordance with the directions set out in the specific manual for each PRO.

A higher percentage of PRO items requiring manual validation may indicate a less patient-friendly PRO format, and is more costly due to the manual labor required. In our sample, the EQ-5D VAS required manual validation about 3 times as often as the other questionnaires, suggesting that the EQ-5D VAS is less useful for a mailed survey in a registry population.

Several methodological problems must be considered when interpreting our results. The EQ-5D index had a bi-modal distribution of data, as previously reported by others (Jansson and Granath 2010), probably due to the EQ-5D algorithm. The implication is that the uncertainties of the results are greater than described by the confidence intervals and p-values, and all the consequences of this may not be known yet. This must be considered when interpreting our results. Our results have high external validity since the distribution of age groups, the sex ratio, diagnoses, and types of prosthesis were similar between our study population and the entire Danish THA population, as well as hip replacement populations seen in other hip registries. Regarding knee arthroplasty, Dunbar (2001) compared properties of the SF-12 and the Oxford knee score in a knee registry setting and found response rates, percentages of fully completed questionnaires, and floor and ceiling effects comparable with our findings from the SF-12 and OHS, suggesting generalizability of our results. We minimized selection bias by randomly selecting patients for inclusion and we tried to achieve equal age and sex composition in the groups.

We conclude that the HOOS, the OHS, the SF-12, and the EQ-5D are all appropriate PROs for administration in a hip registry. We found minor differences between the disease-specific and the generic PROs regarding ceiling and floor effects as well as discarded items. This information may be useful for decision making about the use of particular PROs in a registry-based setting, and other settings of different study design might also benefit from our results.

## Supplementary data

Tables 1 and 2 are available at our website (www.actaorthop.org), identification number 5208.
