# Supplementary material for: Feasibility of 4 patient-reported outcome measures in a registry setting: A cross-sectional study of 6,000 patients from the Danish Hip Arthroplasty Registry
Source: Acta Orthop. 2012 Aug 25;83(4):321–7. doi: 10.3109/17453674.2012.702390 (PMC3427620; doi:10.3109/17453674.2012.702390)
Supplement: Supplementary file 1 [file ORT-1745-3674-83-321-s5208.pdf]

Supplementary article data

Feasibility of 4 patient-reported outcome measures in a registry setting

A cross-sectional study of 6,000 patients from the Danish Hip Arthroplasty Registry

Aksel Paulsen[1], Alma B Pedersen[2], Søren Overgaard[1], and Ewa M Roos[3]

[1]Department of Orthopaedic Surgery and Traumatology, Odense University Hospital, Institute of Clinical Research, University of Southern Denmark, Odense; [2]Department of Clinical Epidemiology, Aarhus University Hospital, Aarhus; [3]Research Unit for Musculoskeletal Function and Physiotherapy, Institute of Sports Science and Clinical Biomechanics, University of Southern Denmark, Odense, Denmark
Correspondence: akselpaulsen@gmail.com

Appendix

Supplementary data

Table 1. Imputing of missing items

PRO	Imputing of missing items	Reference
EQ-5D	No imputing of missing values	User Guide version 2.0
SF-12	Maximum data recovery [a]	QualityMetric Incorporated´s scoring software
HOOS	1 or 2 missing values were substituted with the average value for that subscale. If more than 2 items were omitted, the response was considered invalid and no subscale score was calculated	User's Guide 2003 (updated May 2008)
OHS	1 or 2 missing values were substituted with the average value from all other responses. If more than 2 items were omitted, the response was considered invalid and no overall score was calculated	User Manual version 1.0

[a] QualityMetric Incorporated's scoring software includes an MDE algorithm that enables scoring of PCS and MCS with missing item responses and we used QualityMetric Incorporated's scoring software with missing data estimation method; maximum data recovery (the exact procedure is not described (Ware et al. 2002)), to find percentage of discarded PRO subscales. For all other analyses, we used manual coding with no imputing of missing values.

Table 2. License requirements, fees, and websites

PRO	License requirements and fees	Websites
EQ-5D	A license for the study was obtained from the EuroQol Group. Academic and clinical use of EQ-5D is free of charge if patient numbers are less than 5,000. Where patient numbers exceed 5,000, the EuroQol Group will negotiate with users to collaborate and share data	http://www.euroqol.org
SF-12	A license for the study was obtained from the Medical Outcomes Trust Health Assessment Lab and Quality Metric Incorporated. The fees associated with using SF-12 were altogether 1,569.90 USD (administrative fee, survey reference kit, and scoring software)	http://www.sf-36.org
HOOS	HOOS does not require any licence and is free of charge, even to the medical industry	http://www.koos.nu
OHS	A license for the study and translation was obtained from Isis Innovation. Academic and clinical use of OHS is free of charge	http://www.isis-innovation.com

Ware J E Jr, Kosinski M, Turner-Bowker D M, Gandek B. How to Score Version 2 of the SF-12 Health Survey (With a Supplement Documenting Version 1). 2002. Lincoln, RI: QualityMetric Incorperated.

Open Access - This article is distributed under the terms of the Creative Commons Attribution Noncommercial License which permits any noncommercial use, distribution, and reproduction in any medium, provided the source is credited.
DOI 10.3109/17453674.2012.702390
